# Unveiling Transitions in Disease States: Study of Depressive and Anxiety Symptom Networks over Time

**DOI:** 10.1155/2024/4393070

**Published:** 2024-07-16

**Authors:** Minne Van Den Noortgate, Manuel Morrens, Albert M. Van Hemert, Robert A. Schoevers, Brenda W. J. H. Penninx, Erik J. Giltay

**Affiliations:** ^1^Collaborative Antwerp Psychiatric Research Institute (CAPRI), Faculty of Medicine and Health Sciences, University of Antwerp, Antwerp, Belgium; ^2^Scientific Initiative of Neuropsychiatric and Psychopharmacological Studies (SINAPS), University Psychiatric Centre Duffel, Duffel, Belgium; ^3^Department of Psychiatry, Leiden University Medical Center, Leiden, Netherlands; ^4^University of Groningen, University Center for Psychiatry, Interdisciplinary Centre for Psychopathology and Emotion Regulation, University Medical Center Groningen, Groningen, Netherlands; ^5^Department of Psychiatry, Amsterdam Public Health (Mental Health program) and Amsterdam Neuroscience (Mood, Anxiety, Psychosis, Stress and Sleep Program) Research Institutes, Amsterdam UMC Location Vrije University Amsterdam, Amsterdam, Netherlands; ^6^Health Campus The Hague, Leiden University, The Hague, Netherlands

## Abstract

**Background:**

Major depressive disorder (MDD) and anxiety disorders (AD) have high degrees of comorbidity and show great overlap in symptoms. The analysis of covarying depressive- and anxiety symptoms in longitudinal, sparse data panels has received limited attention. Dynamic time warping (DTW) analysis may help to provide new insights into symptom network properties based on diagnostic- and disease-state stability criteria.

**Materials and Methods:**

In the Netherlands Study of Depression and Anxiety depressive-, anxiety-, and worry symptoms were assessed four or five times over the course of 9 years using self-report questionnaires. The sample included 1,649 participants at baseline, comprising controls (*n* = 360), AD patients (*n* = 158), MDD patients (*n* = 265), and comorbid AD–MDD patients (*n* = 866). With DTW, 1,649 distance matrices were calculated, which yielded symptom networks and enabling comparison of network densities among subgroups.

**Results:**

The mean age of the sample was 41.5 years (standard deviations, 13.2), of whom 66.4% were female. The largest distance was between worry symptoms and physiological arousal symptoms, implicating the most dissimilar dynamics over time. The network density in the groups, from lowest to highest, followed the order: controls, AD, MDD, and comorbid AD–MDD. The comorbid group showed strongly connected mood and cognitive symptoms, which contrasted with the more strongly connected somatic and arousal symptoms in the AD and MDD groups. Groups that showed more transitions in disease states over follow-up, regardless of the diagnoses, had the highest network density compared to more stable states of health or disease (beta for quadratic term = −0.095; *P*  < 0.001).

**Conclusions:**

Symptom networks over time can be visualized by applying DTW methods on sparse panel data. Network density was highest in patients with comorbid anxiety and depressive disorders and those with more instability in disease states, suggesting that a stronger internal connectivity may facilitate “critical transitions” within the complex systems framework.

## 1. Introduction

Comorbidity between psychiatric illnesses is the rule rather than the exception, especially for major depressive disorder (MDD) and anxiety disorders (AD) [1, 2]. In community samples, it has been estimated that 40%–50% of people with a diagnosis of MDD also have a diagnosis of AD at some instance in their lifetime, with around 40% of people getting both diagnoses in the following 12 months [3]. In clinical samples, the reported lifetime comorbidity was even higher, up to 80% [4, 5]. Co-occurrence of the illnesses has been shown to impact disease severity, burden of disease, treatment response, and prognosis, with less favorable results associated with comorbid MDD and AD [2, 6, 7, 8]. Not only does one disorder act as a risk factor for the other, individual symptoms of one disorder are also able to predict symptoms of the other [9].

The latent variable view has been pervasively employed as a model of most psychiatric disorders, i.e., the *common cause framework* [10, 11]. In this view, symptoms like worry or insomnia are seen as a direct result of underlying disorders, such as AD or MDD [11, 12]. Moreover, these symptoms are viewed as equally important and more or less interchangeable, reflected by the common use of unweighted sum scores in MDD and AD rating scales (e.g., inventory of depressive symptomatology (IDS), the Hamilton Rating Scale for Depression (HRSD) or the Beck's anxiety inventory (BAI)) [13, 14]. In recent years, however, network- and complexity theory have come up as alternatives [15]. In these views, mental disorders are not thought of as latent common causes for symptoms but rather as emergent properties that arise out of mutual (often causal) interactions of the intricate web of symptoms, including dynamic positive and negative feedback loops and vicious cycles over different levels [16, 17]. Thus, according to network theory, correlations among symptoms do not come from a shared origin (like an infectious agent leading to all symptoms of the infectious disease) but from temporal and potentially causal relations among symptoms. Complex dynamic systems often show critical thresholds (i.e., tipping points) at which the system shifts abruptly from one state into another [17, 18]. In the field of psychiatry, patients may shift from a healthy to an affective disorder state or vice versa. In several groups of patients, stronger network connections between psychopathological variables were shown when compared to healthy controls or patients in remission [19, 20, 21]. The dynamics of the system of interacting symptoms thus may be more unstable when these units are more closely connected in a denser symptom network. Once a threshold is exceeded and a symptom is (in)activated (e.g., due to adverse stressors or on the reverse due to treatment), the positive feedback associations in such a dense symptom network may propel the system through a phase transition to an alternative stable state of disease or health. As such, resilience may be understood as an emergent state of a system with a more loosely connected network with less reinforcing feedback mechanisms [22].

It can be presumed that nonlinear or nonsimultaneous covariation of symptoms are common in psychiatric disorders. Assumptions such as linear associations and stationarity of data can often not be met in cross-sectional correlational models and advanced multivariate vector autoregression models for time series data [23]. A potential way to map the relationships between symptoms in a dynamic symptom network is dynamic time warping (DTW) [24]. DTW is a statistical method of finding (shape-based) patterns in time-series data. It is used to find optimal alignment in variables that are measured over time. As such, it can find dynamical covariations between variables, even if this covariation is not simultaneous or linear [25], and can quantify the similarity between variables over time.

Most studies utilizing DTW for psychiatric symptom data analysis have included subjects with MDD [26, 27, 28, 29, 30], while two included (also) patients with bipolar disorder [30, 31]. Among them, two reported lower network density to be related to a more favorable treatment response in MDD [26, 27]. In the other studies, network density was not part of their outcome measures. Research questions and included measurements varied greatly, making the outcomes not directly comparable.

In this study, we aim to assess temporal dynamics of depressive, anxiety, and worry symptoms in patients with MDD, AD, or comorbid MDD-AD using DTW network analysis. Symptom networks and network density will be analyzed for (1) the total group, (2) each of the diagnostic subgroups, and (3) in subgroups with different disease states (in)stability. We hypothesize that (a) items concerning single constructs will cluster together (e.g., the 11 items of the Penn State Worry Questionnaire (PSWQ)), (b) network plots based on diagnoses differ in network density and most interconnected symptoms, and (c) chronic disease is linked to a more loose symptom network compared to disease state instability.

The present study will provide important additions to previous research. First, this will be one of the first studies where DTW analysis will be performed on a sparse data panel of psychiatric symptoms, meaning the measurements span over the course of years, with long periods of time in between measurements, in contrast to the use of repeated measurements during the course of days, weeks or a couple of months [26, 27]. This may facilitate a better understanding of long-term symptom dynamics and prognosis. Only one recent study [28] used a sparse data panel comparable to the one used in this study (i.e., measurements every 6 months over a period of 6.5 years), which included only elderly subjects. In contrast, the present study includes adult subjects with MDD, AD, and comorbid MDD-AD, possibly adding valuable insights towards a more dimensional and transdiagnostic approach of psychiatric disorders. In this same light, multiple instruments will be included simultaneously in the analyses, as we will analyze panel data of depressive symptoms, as well as anxiety symptoms, and worry symptoms in all included subjects.

## 2. Materials and Methods

### 2.1. Sample

For the current study, data from the Netherlands Study of Depression and Anxiety (NESDA) was used, an ongoing multisite cohort study, which initially included a cohort of 2,981 adults (18–65 years). Individuals had either a past or current diagnosis of depressive and/or AD (78%) or are healthy controls (22%) [32]. Subjects were excluded from the baseline cohort if they had a primary clinically overt psychotic disorder, bipolar disorder, obsessive–compulsive disorder, or a severe addiction disorder. For more details on NESDA, see Penninx et al. [33]. All subjects provided their written informed consent, and the study protocol was approved by the Ethical Committees of the participating universities.

At baseline, the assessment consisted of sociodemographic variables as well as a diagnostic psychiatric interview. Follow-up assessments were performed at 2, 4, 6, and 9 years. The 9-year follow-up assessment had a response rate of 69.4% (*n* = 2,069) [33]. Subjects were included in the current analyses if they had four or more complete assessments of the IDS, BAI, and PSWQ over the course of the follow-up period, resulting in 1,649 included subjects for the current analyses. Four subgroups were created based on their life-time history of Composite Interview Diagnostic Instrument (CIDI) diagnoses and CIDI diagnoses during the time of follow-up: (a) persons with no history of anxiety or depression, no diagnosis at baseline and no diagnosis during the 9 years of follow-up, named “controls” (*n* = 360); (b) AD patients, i.e., “AD only,” consisting of persons diagnosed with an AD in the past, at baseline and/or during the follow-up period, but never diagnosed with MDD (*n* = 158); (c) “MDD only,” the group of persons diagnosed with MDD in the past, at baseline and/or during the 9 years of follow-up, but never diagnosed with an AD (*n* = 265); (d) “AD–MDD comorbidity,” consisting of persons who had both a diagnosis of AD and MDD in their past, at baseline and/or during the follow-up period (*n* = 866).

### 2.2. Measurements

#### 2.2.1. Psychiatric Diagnoses

The psychiatric diagnoses were assessed by a standardized diagnostic interview (CIDI, version 2.1) at baseline as well as follow-up assessments. The CIDI is used to classify DSM-IV-defined psychiatric diagnoses. For depressive and AD, the instrument has shown high validity, test–retest reliability, and interrater reliability [34]. All AD (i.e., generalized AD, agoraphobia, social phobia, panic disorder with and without agoraphobia) were included, but a diagnosis of MDD was the only included depression diagnosis.

#### 2.2.2. Symptom Severity

The IDS, BAI, and PSWQ were part of the assessment at baseline and all follow-up assessments (2, 4, 6, and 9 years, respectively). The IDS is a questionnaire with a good internal consistency (Cronbach's alpha 0.92–0.94) consisting of 30 items comprising the symptoms of depression as listed in the DSM-IV, as well as anxious, atypical and melancholic symptoms [35], using a 0–3 Likert scale for each item. Anxiety symptoms were measured using the BAI, a 21-item questionnaire (Cronbach's alpha 0.94) using a 0–3 Likert scale for each item [36, 37]. To assess pathological worrying, a brief 11-item version of the PSWQ (Cronbach's alpha 0.84–0.91) was administered using a 0–4 Likert scale [38, 39]. The recall period for both the IDS and BAI was 7 days (“For every symptom, circle to what extent you suffered from this symptom in the past week, including today”); for the PSWQ, this was not specified in the questionnaire (“For each of the following questions, circle to what extent this statement is applicable to you”).

#### 2.2.3. Disease State Stability

A new variable was computed to estimate the (in)stability of the disease-state of each subject. The score was calculated by adding the number of clinical interviews (CIDI) wherein the participants were categorized as having had a diagnosis of AD and/or MDD, either between the current and previous assessment point or, for the baseline interview, a lifetime diagnosis of AD and/or MDD before study inclusion. This yielded a score of 0–5 with the following distribution: 0 (*n* = 360); 1 (*n* = 374); 2 (*n* = 249); 3 (*n* = 252); 4 (*n* = 220), and 5 (*n* = 194). The subjects who scored zero were the control subjects, who had not experienced any affective disorder. A score of 1 implies a relatively stable state of health before or after an active AD and/or MDD diagnosis. A score of 4 or 5 would also imply relative stability, as most of the time, the participant would be in a disease-state (with “disease” being defined by an active diagnosis of AD and/or MDD). Scores of 2 or 3 would imply relative instability, as these scores would include all participants that could have alternated between health- and disease-states, as defined earlier, as well as participants with a longer duration of illness followed or preceded by a longer duration of disorder absence.

### 2.3. Statistical Analysis

Baseline sociodemographic and clinical variables for the whole group, as well as for the diagnostic subgroups, were analyzed and summarized as percentages or as means with standard deviations (SD). Pairwise comparisons between subgroups were performed to assess sociodemographic differences between the groups.

The mean sum scores over time for the IDS, BAI, and PSWQ were calculated according to diagnostic- and disease-state stability subgroups at every assessment point. These trajectories were visualized in a line graph per questionnaire with error bars representing standard errors. Pairwise comparisons between the different subgroups were performed by applying the General Linear Model repeated measures procedure.

The DTW analysis [40] is an algorithm for measuring similarity between two-time series, where each time series is made up of the scores of an individual item over time during the follow-up. The distance between these two short time series yields a dissimilarity measure using the shape-based time-series clustering technique of DTW. This technique aims to find the optimum warping path between the two time-series. All 62 item scores were group-level standardized before the DTW analysis.

**Figure 1:**
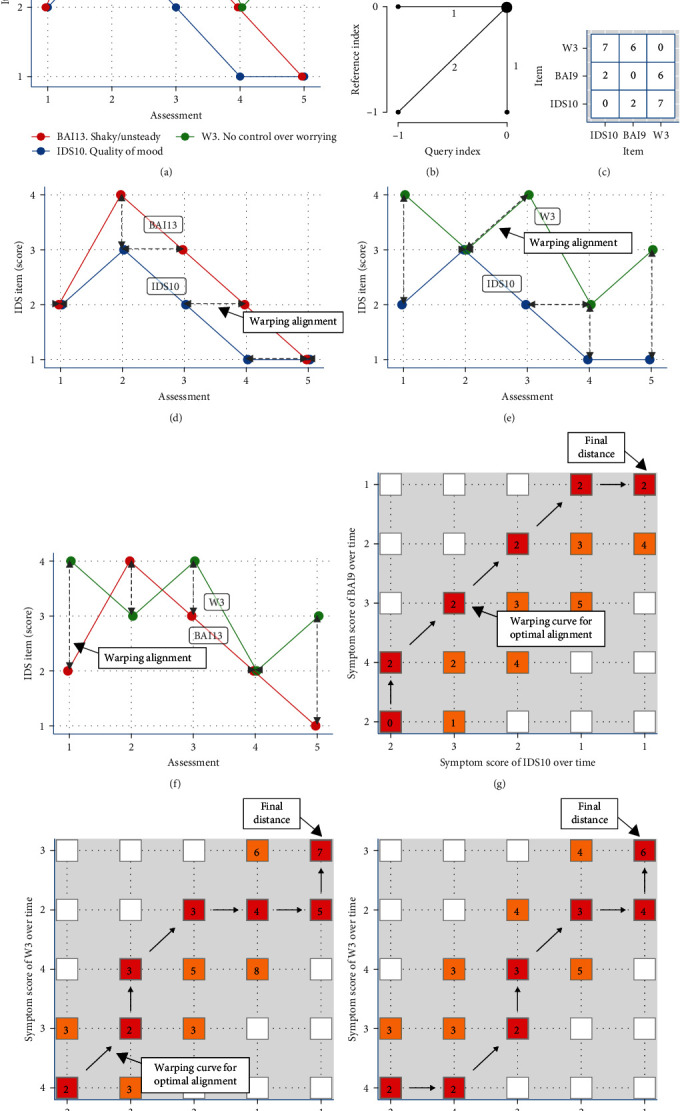
Explanation of the dynamic time warp analysis (DTW) ((a) scores of items IDS10, BA19, W3 over time): a hypothetical example of (unstandardized) scores of individual items over time during the follow-up. The way in which the algorithm traverses through the local cost matrix (LCM) is dictated by the chosen step pattern, in our case, the default “symmetric2” step pattern ((b) step pattern: “symmetric2”). Subparts ((d) scores of IDS10 and BAI13 over time, (e) scores of IDS10 and W3 over time, (f) scores of BAI13 and W3 over time) are a visual representation of each pair of symptom trajectories. We can conclude that the blue and red lines show a more similar route over time (resulting in a DTW distance of only 3) ((g) cost matrix), compared to each distance with the green worry line (resulting in DTW distances of 9 and 6) ((h) cost matrix and (i) cost matrix). Subpart ((c) distance matrix) shows an example of a resulting distance matrix.

Figure 1 shows the explanation of the DTW analysis. The first step involves creating a local cost matrix (LCM), which has 5 × 5 dimensions, as we had 4 or 5 assessments in time (Figures 1(g), 1(h), and 1(i)). In the second step, the DTW algorithm iteratively steps through the LCM, starting in the lower-left corner of the matrix and ending in the upper-right corner, taking steps to minimize the increase in distance (i.e., cost) under the chosen constraint. The constraint in this case was the Sakoe-Chiba band of size one, meaning that the possible steps were limited to a window of one timepoint before and after the current assessment. Given that the time-points in this research are at least 2 years apart, choosing a larger window could possibly lead to erroneous conclusions by aligning item scores very far away in time. The way in which the algorithm traverses through the LCM is furthermore dictated by the chosen step pattern, in this case, the default “symmetric2” step pattern (Figure 1(b)). The step pattern defines the transitions that are allowed to find the minimum-distance path. The “symmetric2” step pattern is widely used, normalizable, and symmetric. This means that the distance from A to B is equal to the distance from B to A, and the distance does not depend on the number of data points from the participant.

In summary, the algorithm will step through the matrix of a pair of items according to the chosen step pattern and constrained by the chosen time window, resulting in the optimal warping path, having the lowest possible cost (i.e., the smallest distance). In other words, it represents the minimum distance that needs to be bridged to let both time series to completely overlap. Thus, items with more similar dynamics over time would result in a small distance (Figures 1(d), 1(e), 1(f), 1(g), 1(h), and 1(i)). We included 62 individual items (30 IDS, 21 BAI, and 11 PSWQ items), resulting in (62^2^−62)/2 = 1,891 distances, yielding 1,649 distance matrices (Figure 1(c)), one for each of the included individuals.

DTW analyses, including all items of the IDS, BAI, and PSWQ, were used to yield network densities and network plots for (1) the total group, and separate network plots for (2) the diagnostic subgroups, and (3) the disease state stability subgroups. This association is stronger when the item scores of an item pair tend to fluctuate in tandem over time. Network plots were also used to visualize the putative differences in symptom networks among subgroups. The network densities were calculated as the mean inverted distances among all 62 items, adjusted for age, sex, and education, using mixed models with a random intercept for participants. These were used to compare diagnostic as well as disease state stability subgroups to one another, which may elucidate the possible connection between diagnoses/disease state stability and network density, as described in detail further on.

**Figure 2:**
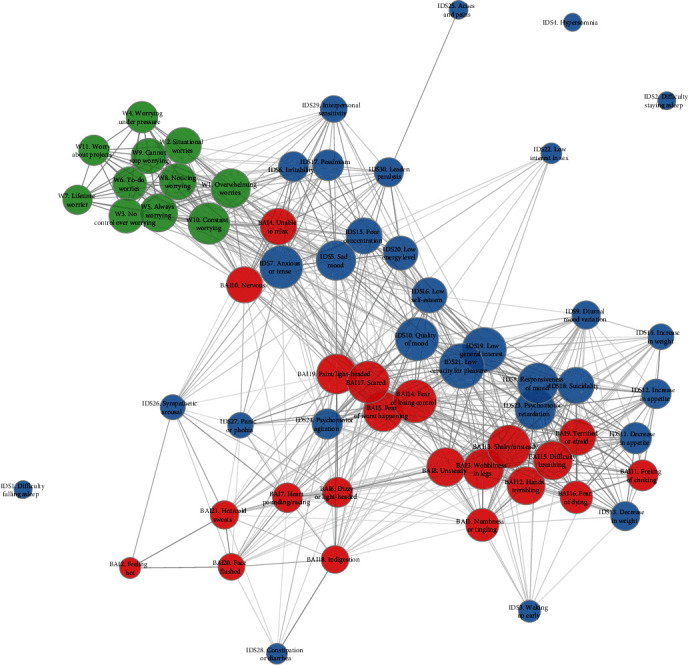
Symptom network structure of the whole group of 1,649 participants. Items are represented as nodes and are color-coded according to the scale they belong to (green = PSWQ, blue = IDS, red = BAI). Item centrality is represented by the size of the node. Edge width is proportional to the effect size estimate. Only statistically significant edges are shown, with a smaller average distance than that of all other pairwise DTW distances (by *t-test for independent samples;P*  < 0.001).

Only the highly significant (*P*  < 0.0001) edges, for which the mean distance was significantly smaller than that of all remaining pairs of distances, were shown in the group-level symptom networks. These analyses were adjusted for the average score of each of the 62 items and for each of the 1,649 participants separately (as item pairs with both scores remaining low and close to zero result in a low distance). Network plots were created using the “qgraph” package for R. In these plots, all items were represented as nodes of the network and color-coded for the scale they belong to. Nodes were connected by straight lines called edges. The connectiveness of a node (i.e., centrality, defined by the mean distance between this node (i.e., item) with all other connected nodes) was represented by the size of the node and the thickness of the edges representing the strength of the association (i.e., beta-coefficient). In network plots of the subgroups, the minimum and maximum edge scores were fixed based on the overall network analysis in order for the edges and edge thickness to be directly comparable among subgroups. Node placement was fixed using the Fruchterman–Reingold algorithm of the “qgraph” *r*-package according to the placement in the whole group (Figure 2). In the network plots of subgroups, the physical distance between the nodes is therefore not a precise measure of pairwise strength any more, whereas only the edge width represents this.

Finally, we computed the network density for all diagnostic and disease stability subgroups, again adjusted for the mean symptom severity, age, sex, and level of education. Network density is a measure for the similarity of symptom dynamics over time in that network. It is the inverse of the average DTW distance in that network. A longer mean DTW distance of a network results in a looser network, thus a lower network density, and vice versa. Given that this calculation makes use of the actual distances and not the significance of the edges, comparisons between differently sized groups are possible. In network plots of the subgroups, the minimum and maximum edge scores were fixed based on the overall network analysis in order for the edges and edge thickness to be directly comparable among subgroups. Node placement was fixed for the same purpose, making the physical distance between nodes in the network plots not a measure of pairwise intercorrelation; it is only the edge width. In order to evaluate network density in relation to diagnostic category and disease state stability, the residuals from a linear multilevel regression analysis, with symptom severity, age, sex, and level of education as fixed factors with a random intercept for participants were calculated and used as the outcome variable in which the disease state stability score and diagnostic categories were used as the independent variable. Both linear and quadratic terms were used for the disease state stability analysis. These adjusted means were plotted with error bars representing standard errors. Group comparisons were performed for the diagnostic subgroups.

We used RStudio (R version 4.2.2; R Foundation for Statistical Computing, Vienna, Austria, 2016. URL: https://www.R-project.org/), with main packages “dtw” (version 1.23.1), DistatisR (version 1.1.1), “parallelDist” (version 0.2.6), “qgraph” (version 1.9.3), and “lme4′ (version 1-1.31).

## 3. Results

### 3.1. Demographic and Clinical Variables at Baseline

Table 1 shows baseline sociodemographic characteristics and mean disease state stability scores of the total sample of included subjects, as well as diagnostic subgroups. The sample consisted of 1,649 subjects, with a mean age of 41.5 years (SD = 13.2), of whom 66.4% were female. The mean disease state stability score of the whole group was 2.1 (SD = 1.7), with the comorbidity subgroup having the highest mean score (3.2, SD = 1.3). The comorbid group included significantly more women than the control group (*P*=0.001) and had a significantly lower level of education than the control group (*P*=0.003). No other significant between-group differences could be identified (all *P*  > 0.05). When comparing the included sample with the nonincluded subjects from the baseline NESDA cohort, statistically significant, yet small, differences could be seen in baseline PSWQ score as well as the included sample having significantly higher levels of education (*Supplementary Table 1*: Comparison of included and nonincluded subjects at baseline).

### 3.2. DTW Analyses in Total Group

In Figure 2, the symptom network plot of the entire group of 1,649 participants is shown. *Supplementary Table 1* shows the distances among item pairs adjusted for the mean item scores within subjects (only positive coefficients are shown with higher values indicating stronger edges), of which the highly significant (*P*  < 0.0001) edges are shown in Figure 2. Of the 1891 edges, 661 (35.0%) were statistically significantly shorter than the distances of all other edges combined (*P*  < 0.0001).

The PSWQ items tended to cluster together much more strongly than those of the BAI or IDS. *Supplementary Figure 1* displays the intercorrelations among symptom pairs across all time points and for all subjects combined. *Supplementary Figure 1* shows the symptom undirected DTW networks, illustrating that clustering is not scale-specific but that the IDS and BAI scales show considerable overlap in terms of symptom dynamics over time within participants.

Items of the BAI and IDS that were connected to the PSWQ were: IDS7 “anxious or tense,” IDS17 “pessimism,” BAI4 “unable to relax,” and BAI10 “nervousness.” There was a large distance, and thus little connectiveness, between physical anxiety symptoms from the BAI and the worry symptoms. The core depressive symptoms tended to be closely connected, such as IDS21 “Low capacity for pleasure” and IDS10 “Quality of mood” as well as IDS5 “Sad mood,” meaning that those symptom trajectories over time were more similar than those of many other symptoms in the network.

The IDS item “sympathetic arousal” was strongly connected to several BAI items (“hot/cold sweats,” “feeling hot,” “heart pounding/racing”). Within the BAI items, somatic symptoms, such as BAI5 “Difficulty breathing,” BAI13 “Shaky/unsteady” and BAI12 “Trembling” also show high interconnectivity. Interestingly, there also was a high degree of connectiveness between IDS18 “suicidality” and those somatic BAI symptoms, as well as some specific IDS items (IDS23 “Psychomotor retardation” and most of the appetite/weight items).

Sleep symptoms were disconnected from many of the rest of the network (IDS4 “hypersomnia,” IDS1 “difficulty falling asleep,” and IDS2 “difficulty staying asleep”) as well as IDS25 “aches and pains,” which is only connected to IDS30 “leaden paralysis,” the only two items in the IDS relating to musculoskeletal symptoms.

The interactive three-dimensional version of the network plot of the distances may aid visualization of the data (which can be downloaded at: https://osf.io/hjn49).

### 3.3. Diagnostic Subgroup Analyses

#### 3.3.1. Sum Scores over Time

**Figure 3:**
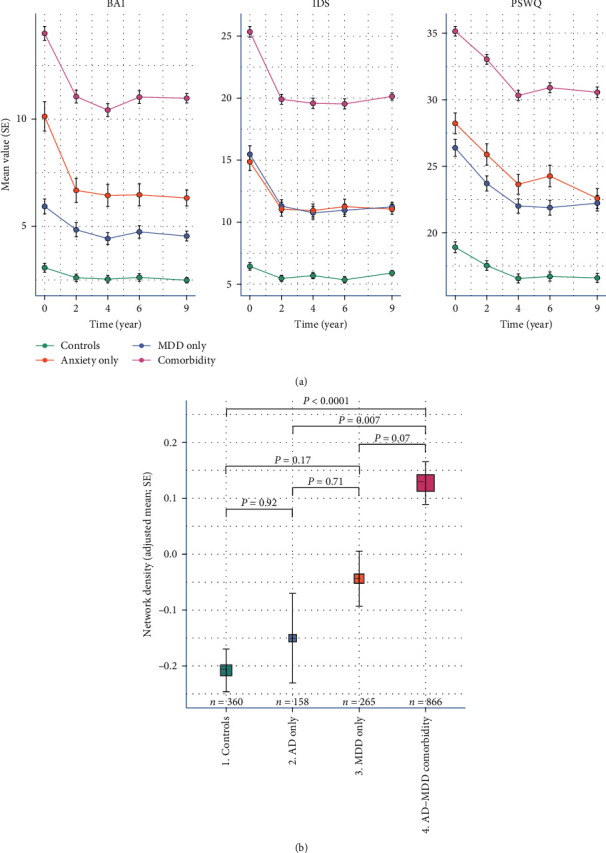
((a) Mean severity over time according to diagnosis groups) Trajectories of mean sum scores of all three questionnaires over time for diagnosis groups over the course of 9 years. Error bars represent the standard error. ((b) Network density according to diagnosis groups) Adjusted mean network density according to diagnosis groups, based on the inverted DTW distances of residuals (adjusted for symptom severity, age, sex, and education with a random slope for participants). Error bars to represent standard errors. The resulting *P*-value from the pairwise comparisons are shown.

The mean sum scores for each of the three questionnaires (IDS, BAI, PSWQ) over time for the diagnostic subgroups are shown in Figure 3(a), and the pairwise comparisons are shown in the table of *Supplementary 2*. The first thing to notice is that across almost all subgroups and all questionnaires, there is an initial drop in mean sum scores from baseline to the first follow-up assessment and then—at the group level—a relatively steady course throughout the rest of the follow-up period as well as a relative stability from baseline in the control group for all three questionnaires. In subgroups with a diagnosis, the AD–MDD comorbidity consistently had the highest scores on all three questionnaires compared to the other subgroups (*P*  < 0.001), but the severity among the “AD only” and “MDD only” groups were similar with regard to the IDS (mean difference 0.298, *Supplementary Table 1*). The “AD only” group scored somewhat higher than “the MDD only” group on both the BAI (mean difference = 1.565) and the PSWQ (mean difference = 1.696), designed to evaluate typical anxiety symptoms, but no statistically significant differences could be observed between those two groups (see table in *Supplementary 2*).

#### 3.3.2. Network Density

The adjusted network densities for the diagnostic subgroups are shown in Figure 3(b). Within the diagnostic subgroups, the AD–MDD comorbidity group has the highest network density and is significantly denser than the control group (*P*  < 0.001) and the “AD only” group (*P*=0.007), but not the “MDD only” group (*P*=0.07). There is no significant difference between the network density of the “AD only” and the “MDD only” groups (*P*=0.71) or any of the other groups compared to one another.

#### 3.3.3. Network Plots and Centrality

**Figure 4:**
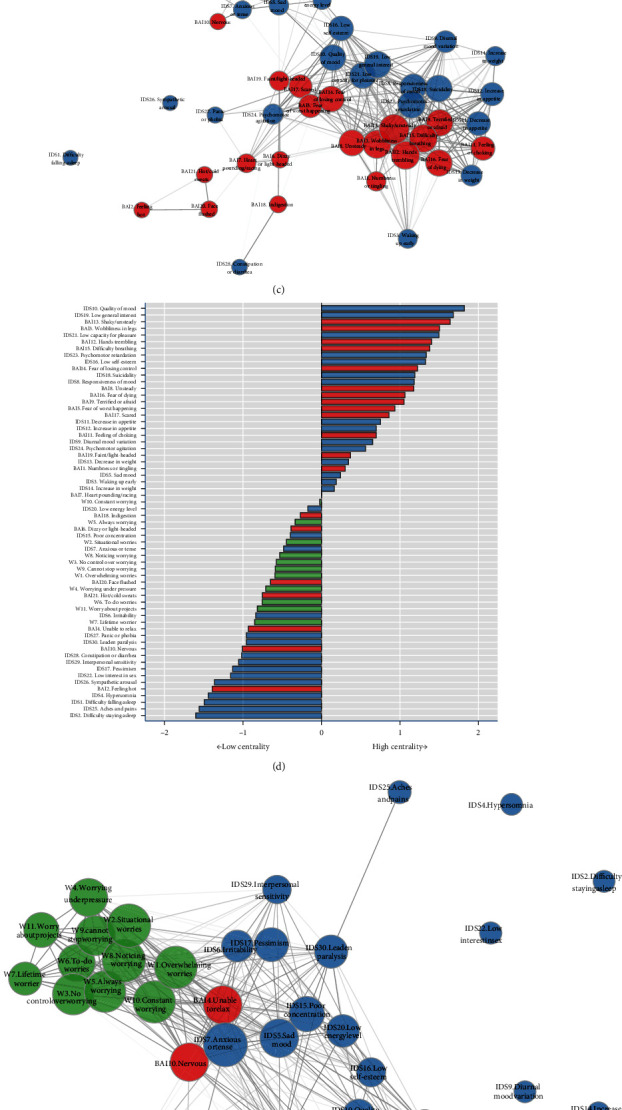
Symptom network structure ((a) symptom network for AD-only (n = 158), (c) symptom network for MDD-only (n = 265), and (e) symptom network for comorbid MDD–AD (n = 866)) and standardized centrality of all items ((b) standardized centrality for AD-only, (d) standardized centrality for MDD-only, and (f) standardized centrality for comorbid MDD–AD) for the diagnostic subgroups. (a, c, and e) Items are represented as nodes and are color-coded according to the scale they belong to (green = PSWQ, blue = IDS, red = BAI). Item centrality is represented by the size of the node. To compare these four graphs, the maximum and minimum values for the edge weights in the graphs were set to similar values (corresponding to the overall analysis by t-test for the independent sample; *P*  < 0.0001). (b, d, and f) Standardized centrality for all items, ranked from high to low centrality, for each of the three subgroups, respectively.

Next, we compared the network plots and item centrality of the diagnosis groups, excluding the control group (Figure 4), in which node placement was fixed. The network plots of the “AD only” and “MDD only” groups showed the most similarities, with only small differences in individual item connectivity but no obvious differences in symptom clusters or overall connectivity. When comparing the “AD–MDD comorbidity group” to both the other patient groups, more distinct differences could be observed. These same (dis)similarities were also obvious when looking at the centrality (Figures 4(b), 4(d), and 4(f)). The higher network density, as mentioned earlier, is visualized in the amount of edges, mainly in the center of the plot that consisted of symptoms that represent core depressive symptoms of the IDS (IDS 10 “quality of mood,” IDS 5 “sad mood,” IDS 20 “low energy level”) as well as cognitive anxiety items (BAI 14 “Fear of losing control,” BAI 5 “Fear of the worst happening,” BAI 17 “Scared”).

However, most of the BAI items, specifically the physical anxiety symptoms, were less connected in the comorbidity network plot. Specific affective anxiety symptoms related to the body (BAI11 “Fear of choking” and BAI 16 “Fear of dying”) were strongly connected to those physical anxiety symptoms and thus also less connected in the comorbid AD–MDD group than can be seen in the mono-diagnosis groups.

The weight and appetite items (separated into “increase” and “decrease” items) of the IDS showed high connectiveness in the “AD only” and “MDD only” subgroups but far less so in the “comorbid AD–MDD” subgroup. IDS 3 “waking up early” is the only sleep symptom that was highly connected in the AD and MDD only plots, mainly to the physical anxiety items of the BAI. Similarly, as mentioned earlier, this IDS item was thus also less connected in the comorbid group. Cognitive IDS items, such as IDS 17 “Pessimism” and IDS 15 “Poor concentration” were more connected in the comorbid group, similar to the worry symptoms and the cognitive anxiety symptoms.

In all three network plots, the sleep symptoms showed very little connectiveness, as can also be seen in the low centrality of those items. The same could be seen for somatic IDS items such as IDS25 “aches and pains” and IDS28 “constipation or diarrhea.”

In summary, the “AD only” and “MDD only” network plots were very similar, whereas in the “comorbid AD–MDD” network plots, there was an increase in general connectivity, mainly reflected by the shift from high connectiveness of physically related BAI and IDS items in the AD and MDD groups to more cognitive symptoms of the PSWQ, BAI, and IDS in the comorbid group.

### 3.4. Disease State (In)Stability Analyses

#### 3.4.1. Sum Scores over Time

**Figure 5:**
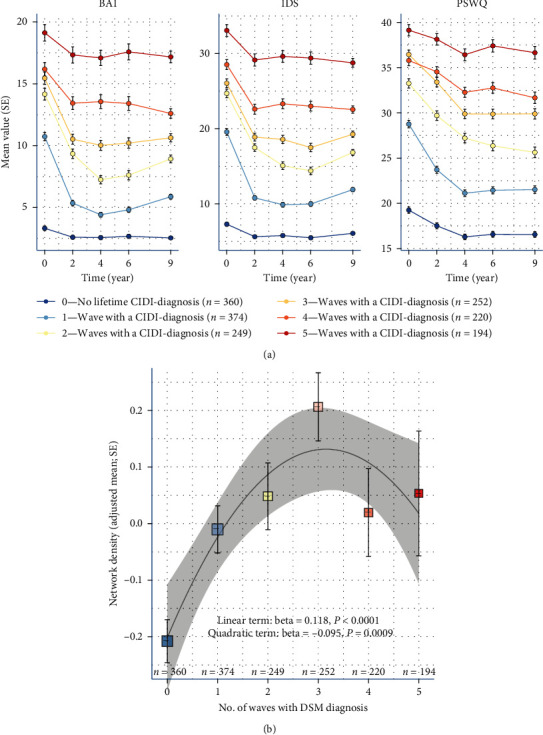
Disease state stability analyses: ((a) mean severity over time according to subgroup of disease state (in)stability) trajectories of mean sum scores of all three questionnaires over time for all different subgroups according to disease state (in)stability over the course of 9 years. Error bars represent the standard error; ((b) network density according to disease state (in)stability) plot of the adjusted mean network density according to disease state (in)stability, based on the inverted DTW distances of residuals (adjusted for symptom severity, age, sex, and education with a random slope for participants). Error bars to represent standard errors. The beta- and *P*-values for both the linear and the quadratic terms of the regression models are shown.

When looking at the mean sum scores in the disease stability analysis, in Figure 5(a), it can be observed that the higher the disease state stability score, the higher the sum scores over time, for all three questionnaires, at all timepoints, including at baseline. The differences were significant (*P*  < 0.001) for all three questionnaires between almost all disease state stability scores (see table in *Supplementary 3*). The most pronounced differences could be seen in the IDS and PSWQ sum scores. The initial drop, from baseline to the first follow-up assessment, is the most pronounced for subjects with a disease stability score of 1 or 2.

#### 3.4.2. Network Density

The adjusted mean network densities for the subjects according to their disease stability score are shown in Figure 5(b). Adding a quadratic term to the linear regression model resulted in a negative beta of −0.095 (*P*-value of 0.0009), indicating a significant fit of this model for the data at hand. Across the six groups, the network density reaches its peak in the most “unstable” group (three waves with active diagnoses) and is lower for more the more “stable” subgroups, with the control group having the lowest density. The results suggest a denser network over time for subjects who go back and forth between having an active diagnosis and being “healthy” and a looser network for subjects who remain relatively stable in their (either presence or absence of) disease state.

## 4. Discussion

In this study, we analyzed panel data of symptom scores using DTW analyses in a sample with and without affective disorders. The (dis)similarities in symptom dynamics over time were visualized in network plots and standardized centrality plots. Network density was higher in patients with comorbid anxiety and depressive disorders and in participants with more instability in disease states, suggesting that a strong internal connectivity may facilitate “critical transitions” within the complex systems framework [17]. These transitions could signify sudden shifts in emotional states, such as moving from a relatively stable mood to a depressive or anxious episode. A strong internal connectivity may allow both activation and inactivation of symptoms to spread more efficiently throughout the network.

DTW analyses on the symptom network of the entire group confirmed the model's face validity. PSWQ items clustered strongly together as expected (given the single construct being assessed) [41], while mood and somatic anxiety symptoms clustered to a lesser extent. Although the PSWQ is regarded as the gold standard questionnaire for worry, relatively few of its items may well capture the essence of pathological worry [42]. The DTW network approach may also signal that, as these items clustered strongly in each group including the control group.

Analyses of diagnostic subgroups revealed largely similar networks for the AD-only and MDD-only groups, whereas the comorbid AD–MDD group showed not only a higher density than the other two groups but also different and stronger connections between cognitive anxiety and mood symptoms. In contrast to recent reviews on cross-sectional symptom networks, we did not find fatigue and sad mood to be the most connected depressive symptoms in the MDD group (i.e., having the highest degree of centrality), although some of the other symptoms concerning quality of mood did score high in that regard [43, 44]. Interestingly, we did find fatigue and sad mood to have higher degrees of centrality in the MDD-AD comorbidity group. One study analyzed both anxiety (i.e., BAI) and depressive symptoms (i.e., Beck Depression Inventory) in 223 patients with major depression [45] and found that in cross-sectional network centrality measures that were very similar to that of our AD and MDD groups, but less comparable to our AD–MDD comorbidity group.

Our analyses also showed that comorbidity of MDD and AD was reflected in higher sum scores on all included questionnaires, which is in line with earlier findings [6, 46]. In a similar population, one study performed prospective (instead of cross-sectional) network analyses using the DTW algorithm, only using IDS symptom data [28], resulting in largely similar network plots and centrality measures. The only two other papers applying DTW analyses on depressive symptoms used different questionnaires (the HRSD and the Comprehensive Psychopathological Rating Scale, respectively), thus making it more difficult to compare network plots and centrality [26, 27]. In the study by Hebbrecht et al. [26], the core mood symptoms did cluster together but showed very low degrees of centrality, whereas Booij et al. [27] did not report centrality measures on the group-level.

Affective disorders can be modeled as a complex dynamic system, as mentioned earlier. It has been hypothesized that denser symptom networks, having strong internal connectivity, are more prone to “tipping” from one state (e.g., health) into another (e.g., disease) and vice versa. In a strongly connected network, the intermediate state may be unstable, because those sets of feedbacks may propel an individual into either a disordered state or a healthy state, with the state in between may be relatively rare [17, 18, 47]. The present study found that patients with comorbid anxiety and depression had a higher network density with more strongly connected cognitive mood and anxiety symptoms such as worrying. This is in line with the theory that more interconnected symptom networks are prone to comorbidity, also represented by the higher degrees of centrality of symptoms in all questionnaires related to both MDD and AD [48]. Another main finding concerned disease state stability. The adjusted network density was higher in those participants who had more instability of disease states over time. There are two previous analyses that applied the DTW method to symptom scores but assessed through routine outcome measuring at weekly or bi-weekly intervals [26, 27]. These studies found that patients who remitted from depression had higher network densities, corroborating our finding that a change in disease state may be associated with a higher network density. This finding contrasts with that of an earlier cross-sectional study, which showed that remitters had a more sparse network than those with persisting MDD [16, 19]. The network plots and density could serve as measures of an individual's susceptibility to transitioning between health and disease states (relapse and remission) [26, 27].

The strengths of this study are the application of the DTW method to study symptom dynamics, the inclusion of a large patient sample with multiple affective disorders, the inclusion of multiple symptom questionnaires, and the long-term follow-up. The DTW algorithm allowed us to process the extensive symptom data of a large patient sample without having to resort to sum scores or other over-simplifications of the available data. This study is the first to include multiple diagnoses and multiple questionnaires for all patients, regardless of their affective diagnosis status, making it possible to transcend the restrictions of classical diagnostic thinking and helping to further explore a more dynamic and dimensional approach towards psychopathology. This study further corroborates the complex dynamic systems framework [17]. In this regard, neatly separating diagnoses (i.e., depressive and AD) seems arbitrary given the similarities in sum scores on all scales, network structures, and density when doing so. This is also in line with contemporary frameworks such as the Hierarchical Taxonomy of Psychopathology that pursues dimensional and transdiagnostic approaches [49, 50].

Nonetheless, the limitations of this study should be considered. First, we were only able to include around 55% of the initial subjects included in the NESDA study. It is possible that the disease course of the subjects that dropped out of the study is markedly different, than those we did include, which could influence the generalizability of the results. Second, we did not include data on pharmacological or other interventions in our analyses, which could all affect symptom networks and network properties. Third, DTW analyses have not yet been extensively applied to sparse panel data, and no robust guidelines have been reported on the effect of the time intervals between assessments or the number of assessments needed. We mostly adopted the default settings of the DTW algorithm, based on prior research, but further research is necessary to investigate the optimal settings (e.g., Sakoe-Chiba bandwidth, time intervals, number of assessments) for specific types of data in psychiatric research. The analyses performed are sensitive to changes over time. Two out of the three questionnaires (IDS and BAI) included a time indicator (i.e., “in the last 2 weeks”), but the PSWQ did not. It could be argued that this could lead to more stability in the PSWQ items and skew the results. However, when looking at the sum scores over time, this does not seem to be the case, as there is a clear drop in average PSWQ scores from baseline, similar to the other two included questionnaires. Furthermore, disease state stability was estimated by counting the number of assessments where the subject had an active DSM diagnosis between the last assessment and the current one. However, since this is not the most precise measure of disease state stability, some measurement errors may have likely led to an underestimation of the effect with network density. Moreover, many participants from the control group had minimal changes in item scores, with most scoring zero. DTW relies on changes over time, and therefore, the data from this group were considered less informative for establishing connections between symptom dynamics. Finally, it should be noted that our findings also depended on the questionnaires that were included in the analyses. By adding the PSWQ, we increased the number of cognitive anxiety symptoms and the weight of worrying as a symptom on the connectivity and, thus, the density of the networks. Given that we previously reported a shift towards more connected cognitive symptoms in the comorbid group, the higher connectivity of the PSWQ items could have contributed to the higher network density found in this group.

In conclusion, this study offered further insight into the complex nature of the relationship between MDD and AD as well as novel insights into the specific symptom dynamics. It also added new information to the discussion on the clinical relevance of symptom network density in relation to disease state stability and comorbidity. DTW may prove to be a promising methodology to provide insight into the complex interactions of psychiatric symptoms, transcending diagnoses, in order to facilitate a more dimensional approach to psychiatric disorders and their prognoses and aid in a more precise and personalized approach to psychiatric medicine. More research should be done to optimize prospective network analyses and uncover the clinical relevance of symptom networks and network density in clinical and outpatient patient samples, considering follow-up time, number of assessments, time intervals between assessments, treatments, and treatment outcomes.

## Figures and Tables

**Table 1:** Baseline characteristics of the total sample of included subjects, as well as the diagnostic subgroups separately.

	Total sample (*n* = 1,649)	Controls (*n* = 360)	MDD only (*n* = 265)	AD only (*n* = 158)	AD/MDD comorbidity (*n* = 866)
Demographic characteristics					
Age at baseline in years (mean; SD)	41.5 (13.2)	41.6 (14.7)	41.5 (13.4)	40.8 (13.6)	41.7 (12.3)
Female (%)	66.4	58.1	65.7	65.8	70.1
Level of education (%)					
Basic	3.3	3.1	1.9	2.5	3.9
Intermediate	55.4	47.8	56.2	57.6	58.0
High	41.3	49.2	41.9	39.9	38.1
Disease state stability score (mean, SD)	2.1 (1.70)	0.00 (0.00)	1.5 (0.8)	1.9 (1.3)	3.2 (1.3)

## Data Availability

The data that support the findings of this study are available from the NESDA consortium (www.nesda.nl), but restrictions apply to the availability of these data, which were used with specific permission for the current study and are not publicly available. Data are, however, available from the authors upon reasonable request and with permission of the NESDA consortium.

## Supplementary Materials

Supplementary 1 Figure 1: displays the intercorrelations among symptom pairs across all time points and for all subjects combined. Figure 2: shows the symptom undirected DTW networks, illustrating that clustering is not scale-specific but that the IDS and BAI scales show considerable overlap in terms of symptom dynamics over time within participants. Table 1: shows the distances among item pairs adjusted for the mean item scores within subjects, of which the highly significant (*P* < 0.0001) edges are shown in Figure 2. Table 2: shows the baseline demographic characteristics and sum scores of the total sample and the included and non-included subjects separately.

Supplementary 2 Results of the generalized linear model of repeated measures of sum scores over time of (a) the PSWQ, (b) the BAI, and (c) the IDS for the diagnostic subgroups showing the overall F-value of between-subjects contrast, mean difference (MD) and standard error (SE).

Supplementary 3 Results of the generalized linear model of repeated measures of sum scores over time of the PSWQ, the BAI, and the IDS comparing subsequent disease state stability scores, showing the overall *F*-value of between-subjects contrast, mean difference (MD), and standard error (SE).
