# Supplementary material for: Unveiling Transitions in Disease States: Study of Depressive and Anxiety Symptom Networks over Time
Source: Depress Anxiety. 2024 Jul 16;2024:4393070. doi: 10.1155/2024/4393070 (PMC11918905; doi:10.1155/2024/4393070)
Supplement: Supplementary 1 — Figure 1: displays the intercorrelations among symptom pairs across all time points and for all subjects combined. Figure 2: shows the symptom undirected DTW networks, illustrating that clustering is not scale-specific but that the IDS and BAI scales show considerable overlap in terms of symptom dynamics over time within participants. Table 1: shows the distances among item pairs adjusted for the mean item scores within subjects, of which the highly significant (P < 0.0001) edges are shown in Figure 2. Table 2: shows the baseline demographic characteristics and sum scores of the total sample and the included and non-included subjects separately. [file 4393070.f1.pdf]

Supplementary Materials

Supplementary Figure 1: Density plot of intercorrelation coefficients

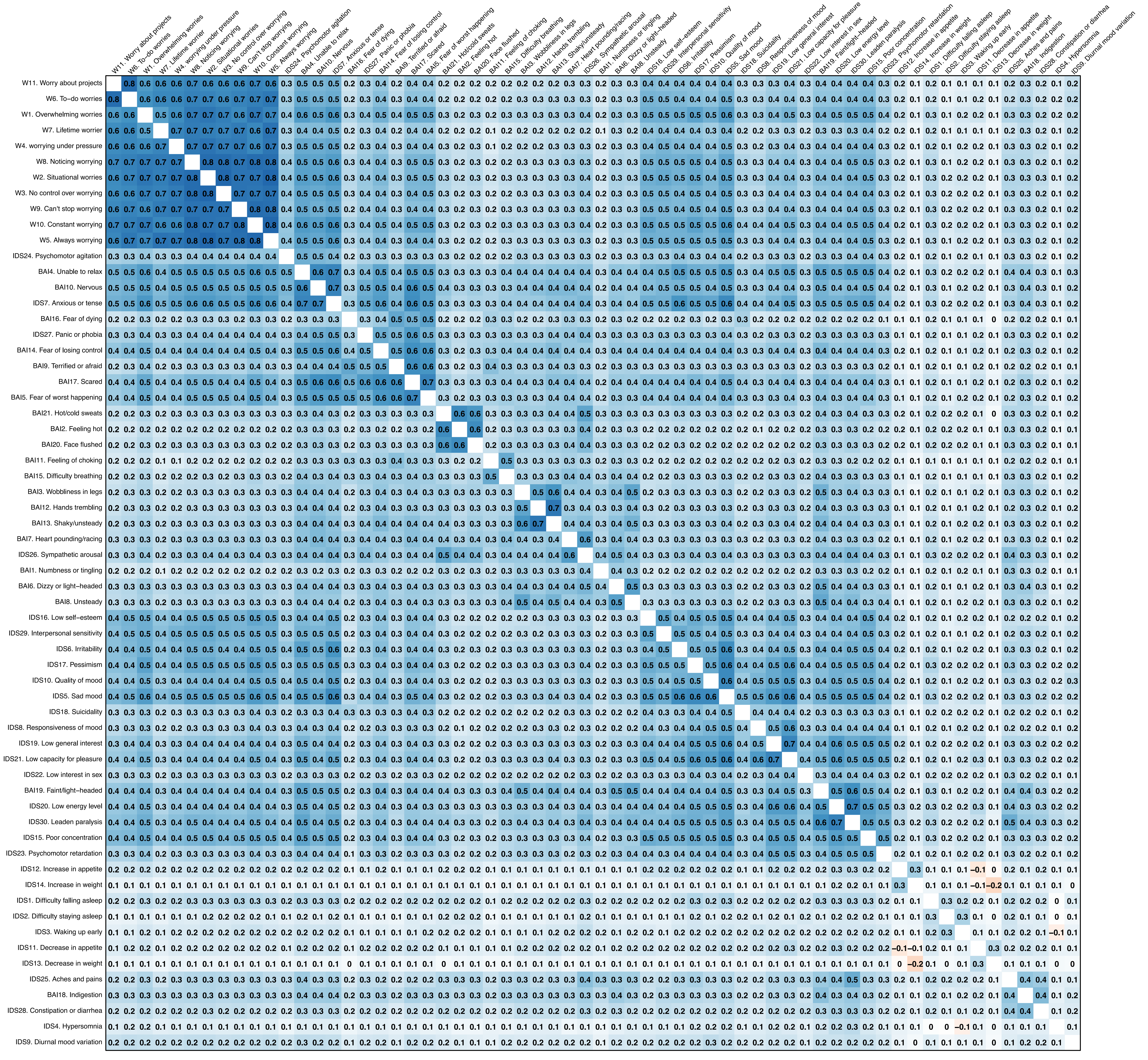

Supplementary Figure 2: Symptom undirected DTW networks

A. Symptom network, color coded according to scales

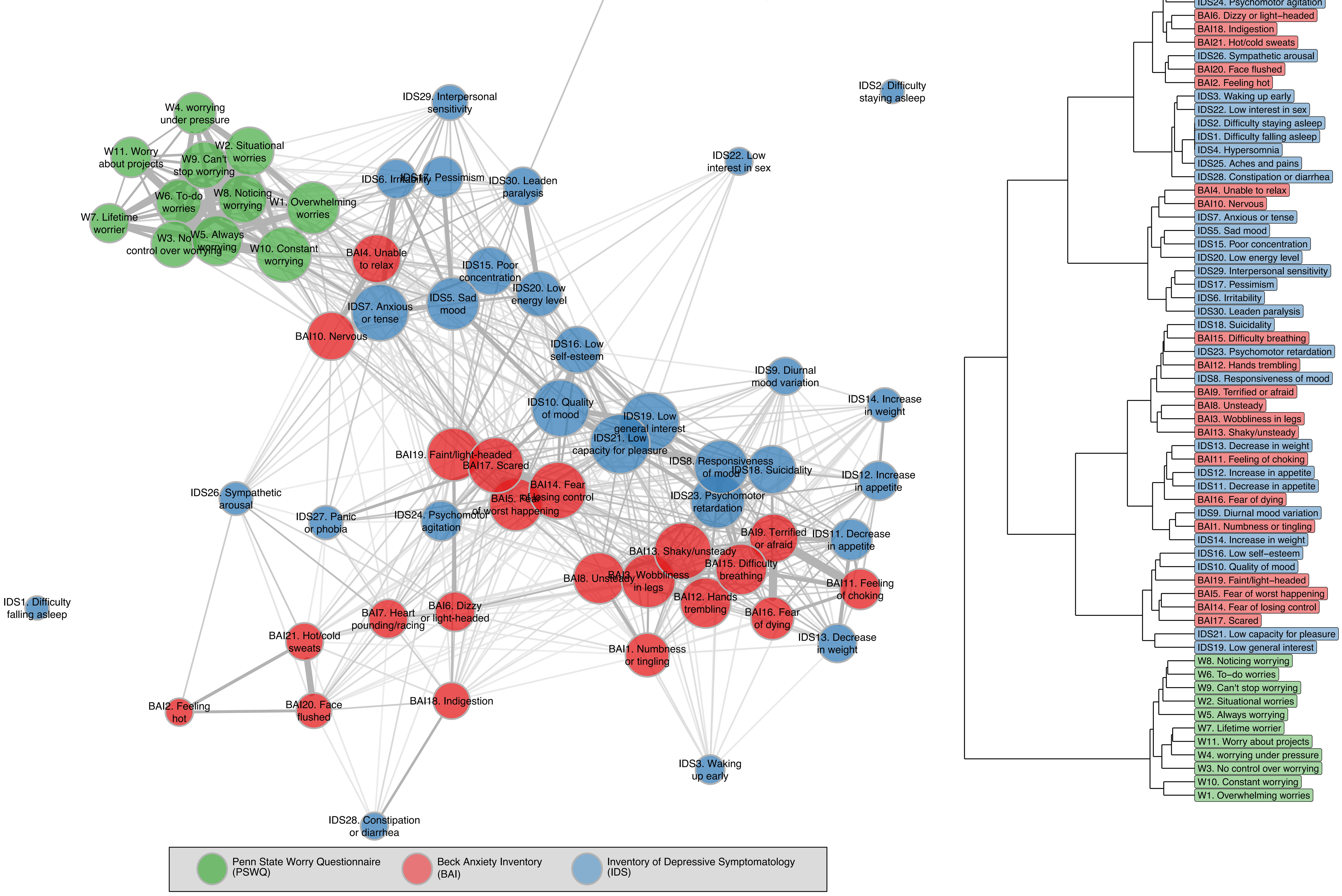

B. Symptom network, color coded according cluster analysis

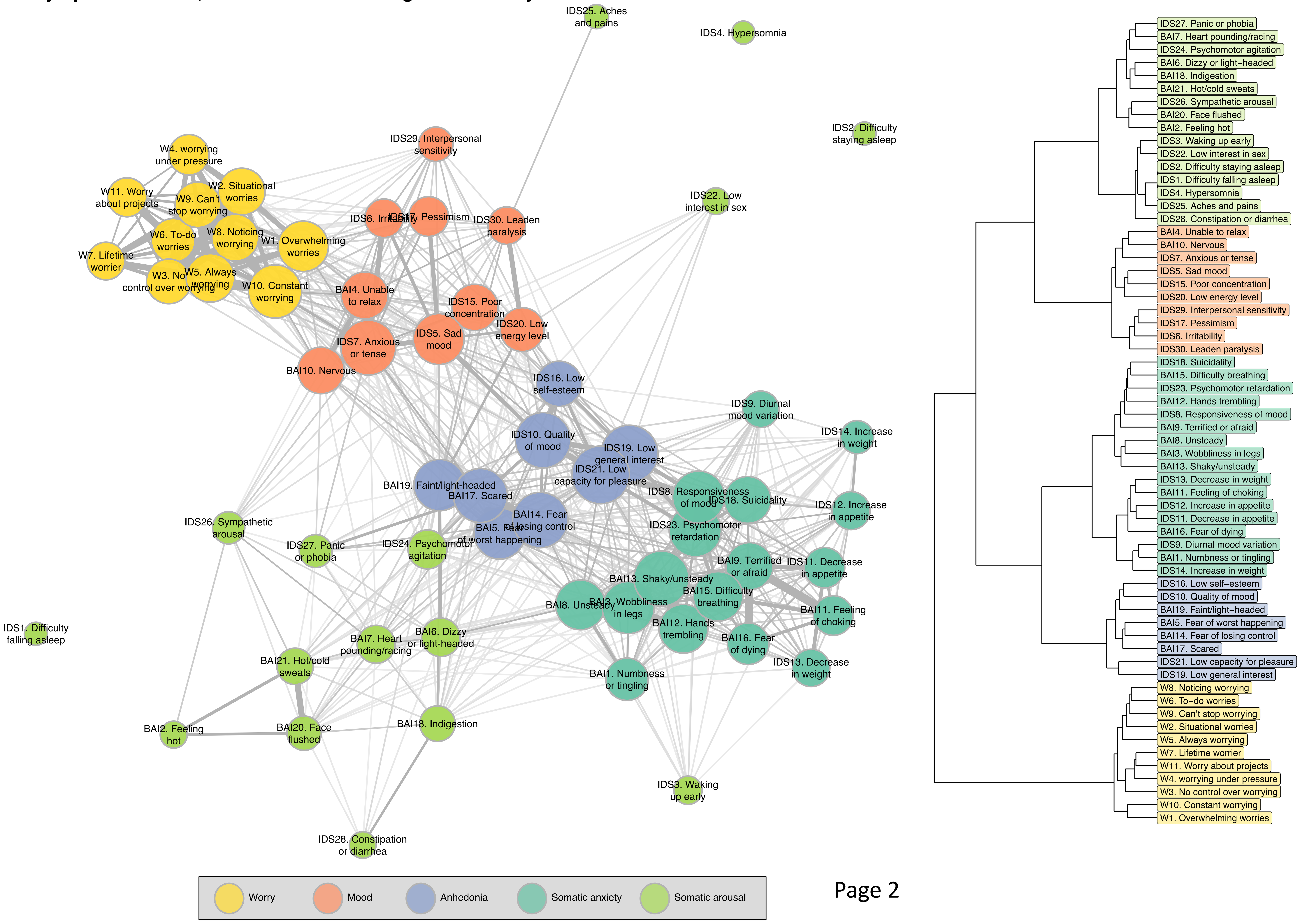

Supplementary Table 1: Adjusted paired distance among items

Undirected distance matrix (n=1,649) using Dynamic Time Warp (DTW)

	IDS1. Difficulty falling asleep	IDS2. Difficulty staying asleep	IDS3. Waking up early	IDS4. Hypersomnia	IDS5. Sad mood	IDS6. Irritability	IDS7. Anxious or tense	IDS8. Responsiveness of mood	IDS9. Diurnal mood variation	IDS10. Quality of mood	IDS11. Decrease in appetite	IDS12. Increase in appetite	IDS13. Decrease in weight	IDS14. Increase in weight	IDS15. Poor concentration	IDS16. Low self-esteem	IDS17. Pessimism	IDS18. Suicidality	IDS19. Low general interest	IDS20. Low energy level	IDS21. Low capacity for pleasure	IDS22. Low interest in sex	IDS23. Psychomotor retardation	IDS24. Psychomotor agitation	IDS25. Aches and pains	IDS26. Sympathetic arousal	IDS27. Panic or phobia	IDS28. Constipation or diarrhea	IDS29. Interpersonal sensitivity	IDS30. Leaden paralysis	BAI1. Numbness or tingling	BAI2. Feeling hot	BAI3. Wobbliness in legs	BAI4. Unable to relax	BAI5. Fear of worst happening	BAI6. Dizzy or light-headed	BAI7. Heart pounding/racing	BAI8. Unsteady	BAI9. Terrified or afraid	BAI10. Nervous	BAI11. Feeling of choking	BAI12. Hands trembling	BAI13. Shaky/unsteady	BAI14. Fear of losing control	BAI15. Difficulty breathing	BAI16. Fear of dying	BAI17. Scared	BAI18. Indigestion	BAI19. Faint/light-headed	BAI20. Face flushed	BAI21. Hot/cold sweats	W1. Overwhelming worries	W2. Situational worries	W3. No control over worrying	W4. worrying under pressure	W5. Always worrying	W6. To-do worries	W7. Lifetime worrier	W8. Noticing worrying	W9. Can't stop worrying	W10. Constant worrying	W11. Worry about projects
IDS1. Difficulty falling asleep		0	0	0	0	0	0	0	0	0	0	0	0	0	0	0	0	0	0	0	0	0	0	0	0	0	0	0	0	0	0	0	0	0	0	0	0	0	0	0	0	0	0	0	0	0	0	0	1.35	0	0	0	0	0	0	0	0	0	0	0	0	0
IDS2. Difficulty staying asleep	0		0	0	0	0	0	0	0	0	0	0	0	0	0	0	0	0	0	0	0	0	0	0	0	0	0	0	0	0	0	0	0	0	0	0	0	0	0	0	0	0	0	0	0	0	0	0	0	0	0	0	0	0	0	0	0	0	0	0	0	0
IDS3. Waking up early	0	0		0	0	0	0	7.53***	2.63*	2.85*	2.61*	0.75	4.28***	5.68***	0	0.52	0	5.15***	6.45***	0	6.53***	0	4.61***	0	0	0	0	0	0	0	6.43***	0	4.10***	0	0.80	0	0	6.02***	0.93	0	0	3.46**	4.65***	1.50	3.11*	0.67	0	0	0	0	0	0	0	0	0	0	0	0	0	0	0	0
IDS4. Hypersomnia	0	0	0		0	0	0	0	0	0	0	0	0	0	0	0	0	0	0	0	0	0	0	0	0	0	0	0	0	0	0	0	0	0	0	0	0	0	0	0	0	0	0	0	0	0	0	0	0	0	0	0	0	0	0	0	0	0	0	0	0	0
IDS5. Sad mood	0	0	0	0		20.34***	23.00***	6.95***	0	20.68***	0	0	0	0	20.62***	13.34***	21.49***	3.63**	13.89***	17.80***	15.77***	4.24***	2.74*	5.84***	0	3.57**	4.52***	0	11.62***	10.29***	0	0	0	12.77***	8.78***	4.55***	2.10	0	0	12.32***	0	0	0	7.69***	0	0	10.06***	0.90	13.47***	0	1.04	13.11***	7.49***	5.56***	2.66*	8.42***	6.50***	0	6.95***	6.24***	13.92***	2.17
IDS6. Irritability	0	0	0	0	20.34***		22.26***	0	0	6.98***	0	0	0	0	12.45***	3.10*	13.23***	0	3.25*	10.88***	3.57**	0	0	1.66	0	3.99***	0	0	13.72***	9.96***	0	0	0	15.33***	2.21	0.93	0	0	0	13.36***	0	0	0	2.91*	0	0	1.84	0	7.66***	0	0	9.28***	3.91***	3.07*	1.52	6.20***	5.97***	0	4.04***	4.62***	10.83***	1.21
IDS7. Anxious or tense	0	0	0	0	23.00***	22.26***		0	0	11.02***	0	0	0	0	16.48***	7.67***	16.43***	0	4.73***	13.46***	6.97***	0	0	6.43***	0	10.29***	11.45***	0	13.01***	10.65***	0	0	0	22.74***	12.41***	5.26***	5.63***	0	0	26.99***	0	0	0	11.71***	0	0	16.60***	2.79*	10.58***	0	1.42	15.64***	10.83***	9.79***	7.45***	11.93***	9.47***	2.79*	10.75***	9.27***	18.35***	6.19***
IDS8. Responsiveness of mood	0	0	7.53***	0	6.95***	0	0		9.27***	17.42***	15.10***	13.19***	12.67***	7.00***	2.07	11.36***	0	22.52***	21.05***	3.85**	24.95***	0.29	25.19***	4.40***	0	0	0	0	0	0	9.97***	0	14.50***	0	7.13***	0	0	10.23***	15.68***	0	12.61***	16.07***	18.64***	10.60***	18.56***	11.73***	7.51***	0	4.70***	0	0	0	0	0	0	0	0	0	0	0	0	0
IDS9. Diurnal mood variation	0	0	2.63*	0	0	0	0	9.27***		9.30***	5.89***	5.51***	5.96***	7.25***	0	4.99***	0	9.01***	10.35***	0	9.14***	0	9.16***	4.36***	0	0	0	0	0	0	5.19***	0	9.96***	0	3.82**	0	0	8.73***	6.11***	0	3.51**	7.87***	8.75***	6.91***	8.38***	5.10***	4.18***	0.03	1.73	0	0	0	0	0	0	0	0	0	0	0	0	0
IDS10. Quality of mood	0	0	2.85*	0	20.68***	6.98***	11.02***	17.42***	9.30***		3.14*	4.15***	1.46	4.24***	12.06***	21.89***	6.95***	12.72***	23.30***	12.18***	24.87***	5.79***	13.44***	15.08***	0	0	5.00***	2.10	3.67**	5.43***	6.87***	0	8.50***	6.86***	15.12***	5.16***	4.96***	9.99***	5.00***	5.85***	0.70	6.79***	9.31***	17.98***	6.75***	1.06	16.91***	5.78***	13.37***	4.25***	6.84***	6.57***	3.92***	2.34	0.33	3.43**	0.89	0	3.58**	1.26	6.71***	0
IDS11. Decrease in appetite	0	0	2.61*	0	0	0	0	15.10***	5.89***	3.14*		6.56***	20.06***	2.10	0	2.67*	0	11.62***	8.39***	0	8.05***	0	15.90***	0	0	0	0	0	0	0	5.69***	0	10.51***	0	0.24	0	0	6.71***	20.27***	0	17.55***	12.35***	14.35***	3.87**	12.62***	15.04***	0.11	0	0	0	0	0	0	0	0	0	0	0	0	0	0	0
IDS12. Increase in appetite	0	0	0.75	0	0	0	0	13.19***	5.51***	4.15***	6.56***		8.31***	18.59***	0	3.32**	0	10.89***	9.16***	0	6.28***	0	15.04***	0	0	0	0	0	0	0	4.45***	0	8.06***	0	0	0	0	3.83**	13.70***	0	12.61***	8.94***	10.77***	2.14	12.76***	12.03***	0	0	0	0	0	0	0	0	0	0	0	0	0	0	0	0
IDS13. Decrease in weight	0	0	4.28***	0	0	0	0	12.67***	5.96***	1.46	20.06***	8.31***		0	0	0.08	0	11.59***	5.86***	0	3.91***	0	12.85***	0	0	0	0	0	0	0	5.83***	0	9.34***	0	0	0	0	5.85***	10.57***	0	9.52***	10.82***	12.80***	2.28	12.03***	9.77***	0	0	0	0	0	0	0	0	0	0	0	0	0	0	0	0
IDS14. Increase in weight	0	0	5.68***	0	0	0	0	7.00***	7.25***	4.24***	2.10	18.59***	0		0	2.76*	0	5.95***	8.83***	0	6.09***	0	7.65***	0	0	0	0	0	0	0	7.11***	0	7.22***	0	1.58	0	0	5.74***	5.18***	0	4.55***	6.02***	6.69***	3.25*	7.80***	3.84**	0.42	0	0	0	0	0	0	0	0	0	0	0	0	0	0	0
IDS15. Poor concentration	0	0	0	0	20.62***	12.45***	16.48***	2.07	0	12.06***	0	0	0	0		12.15***	12.82***	0	13.24***	21.37***	11.70***	5.67***	7.78***	6.90***	0	5.33***	3.14*	0.94	12.22***	12.58***	0	0	0	13.31***	5.73***	5.78***	1.38	0.41	0	11.00***	0	0	0	6.96***	0	0	6.91***	2.42	13.29***	0	1.87	9.98***	4.77***	3.65**	2.72*	5.48***	5.89***	0	4.12***	4.66***	9.65***	3.01*
IDS16. Low self-esteem	0	0	0.52	0	13.34***	3.10*	7.67***	11.36***	4.99***	21.89***	2.67*	3.32**	0.08	2.76*	12.15***		4.82***	11.41***	18.62***	7.94***	19.10***	3.34**	9.82***	12.07***	0	0	3.08*	0	10.48***	3.29*	3.69**	0	4.76***	3.89**	14.62***	1.95	3.43**	7.12***	3.87**	4.23***	0	4.91***	6.00***	16.69***	3.54**	0.09	14.54***	2.55	9.98***	0.32	2.50	6.05***	4.60***	2.32	0.71	4.41***	3.19*	0	4.18***	1.64	7.91***	2.07
IDS17. Pessimism	0	0	0	0	21.49***	13.23***	16.43***	0	0	6.95***	0	0	0	0	12.82***	4.82***		0	4.84***	10.36***	5.69***	0.22	0	0	0	5.38***	0	0	9.20***	10.43***	0	0	0	10.81***	1.51	0	0	0	0	9.34***	0	0	0	0	0	0	3.18*	0	3.75**	0	0	13.64***	6.74***	5.23***	1.15	7.67***	4.82***	0	5.53***	6.08***	11.79***	1.13
IDS18. Suicidality	0	0	5.15***	0	3.63**	0	0	22.52***	9.01***	12.72***	11.62***	10.89***	11.59***	5.95***	0	11.41***	0		14.88***	0	15.75***	0	19.09***	2.70*	0	0	0	0	0	0	8.18***	0	11.11***	0	7.76***	0	0	8.93***	14.87***	0	9.81***	12.72***	15.21***	9.06***	15.67***	16.76***	7.71***	0	1.29	0	0	0	0	0	0	0	0	0	0	0	0	0
IDS19. Low general interest	0	0	6.45***	0	13.89***	3.25*	4.73***	21.05***	10.35***	23.30***	8.39***	9.16***	5.86***	8.83***	13.24***	18.62***	4.84***	14.88***		14.75***	34.46***	7.88***	21.03***	9.67***	0	0	2.11	3.56**	1.83	6.60***	11.33***	0	12.35***	4.72***	12.27***	5.47***	3.79**	14.74***	7.48***	1.26	4.81***	11.50***	13.07***	17.19***	10.45***	4.07***	12.54***	5.97***	13.36***	1.21	3.99***	2.73*	0.57	0	0	0	0	0	0.15	0	2.37	0
IDS20. Low energy level	0	0	0	0	17.80***	10.88***	13.46***	3.85**	0	12.18***	0	0	0	0	21.37***	7.94***	10.36***	0	14.75***		13.89***	9.01***	7.00***	3.17*	1.98	7.22***	3.62**	4.01***	6.78***	23.02***	0	0.11	2.23	10.66***	3.95***	7.51***	3.10*	4.67***	0	8.17***	0	0	1.83	5.72***	0.04	0	5.64***	4.94***	18.22***	1.08	4.27***	6.61***	0.62	0.11	0	0.30	1.41	0	0.71	0.02	5.56***	0
IDS21. Low capacity for pleasure	0	0	6.53***	0	15.77***	3.57**	6.97***	24.95***	9.14***	24.87***	8.05***	6.28***	3.91***	6.09***	11.70***	19.10***	5.69***	15.75***	34.46***	13.89***		7.96***	20.18***	10.66***	0	0	1.99	1.00	1.27	5.96***	11.00***	0	12.43***	6.56***	12.88***	5.34***	4.22***	14.70***	7.59***	2.01	4.37***	11.37***	13.44***	17.14***	11.04***	3.71**	14.19***	5.02***	12.28***	1.30	4.27***	4.22***	1.13	0	0	0	0	0	0.28	0	4.11***	0
IDS22. Low interest in sex	0	0	0	0	4.24***	0	0	0.29	0	5.79***	0	0	0	0	5.67***	3.34**	0.22	0	7.88***	9.01***	7.96***		1.96	0	0	0	0	0.44	0	1.42	0	0	0	0	1.60	2.05	0.59	0	0	0	0	0	0	0.10	0	0	2.02	0	4.68***	0.20	0.12	0	0	0	0	0	0	0	0	0	0	0
IDS23. Psychomotor retardation	0	0	4.61***	0	2.74*	0	0	25.19***	9.16***	13.44***	15.90***	15.04***	12.85***	7.65***	7.78***	9.82***	0	19.09***	21.03***	7.00***	20.18***	1.96		4.84***	0	0	0	0	0	2.47	10.80***	0	16.26***	0	5.45***	1.43	0	14.46***	14.58***	0	12.12***	17.04***	19.52***	10.53***	19.60***	11.04***	5.06***	0.49	6.40***	0	0.57	0	0	0	0	0	0	0	0	0	0	0
IDS24. Psychomotor agitation	0	0	0	0	5.84***	1.66	6.43***	4.40***	4.36***	15.08***	0	0	0	0	6.90***	12.07***	0	2.70*	9.67***	3.17*	10.66***	0	4.84***		0	0	4.04***	0	0	0	4.70***	0	7.87***	7.13***	12.43***	3.47**	6.56***	6.68***	0.31	6.02***	0	4.32***	5.51***	12.36***	3.02*	0	13.11***	5.78***	8.82***	4.19***	4.81***	0	0	0	0	0	0	0	0	0	1.27	0
IDS25. Aches and pains	0	0	0	0	0	0	0	0	0	0	0	0	0	0	0	0	0	0	0	1.98	0	0	0	0		1.78	0	0	0	12.48***	0	0	0	0	0	0	0	0	0	0	0	0	0	0	0	0	0	0	0	0	0	0	0	0	0	0	0	0	0	0	0	0
IDS26. Sympathetic arousal	0	0	0	0	3.57**	3.99***	10.29***	0	0	0	0	0	0	0	5.33***	0	5.38***	0	0	7.22***	0	0	0	0	1.78		4.37***	0	0	7.45***	0	10.60***	0	5.99***	0	8.81***	14.01***	0	0	7.90***	0	0	0	0	0	0	0.20	1.58	3.96***	5.99***	11.09***	1.23	0	0	0	0	0	0	0	0	2.37	0
IDS27. Panic or phobia	0	0	0	0	4.52***	0	11.45***	0	0	5.00***	0	0	0	0	3.14*	3.08*	0	0	2.11	3.62**	1.99	0	0	4.04***	0	4.37***		0	0.08	0	0	0	0.10	2.27	12.76***	8.85***	8.81***	1.21	2.40	8.78***	0	0	1.05	13.46***	0	0	18.69***	3.46**	8.01***	3.81**	4.38***	0.23	0	0	0	0	0	0	0	0	0.93	0
IDS28. Constipation or diarrhea	0	0	0	0	0	0	0	0	0	2.10	0	0	0	0	0.94	0	0	0	3.56**	4.01***	1.00	0.44	0	0	0	0	0		0	0	0.42	0	0.58	0	0.92	4.62***	3.61**	0.63	0	0	0	0	0	1.20	0	0	2.33	17.34***	7.20***	4.49***	4.23***	0	0	0	0	0	0	0	0	0	0	0
IDS29. Interpersonal sensitivity	0	0	0	0	11.62***	13.72***	13.01***	0	0	3.67**	0	0	0	0	12.22***	10.48***	9.20***	0	1.83	6.78***	1.27	0	0	0	0	0	0.08	0		7.07***	0	0	0	10.39***	0.93	0	0	0	0	11.37***	0	0	0	2.81*	0	0	2.86*	0	3.22*	0	0	7.95***	5.62***	3.49**	3.01*	4.34***	4.70***	0	4.09***	3.70**	8.31***	1.12
IDS30. Leaden paralysis	0	0	0	0	10.29***	9.96***	10.65***	0	0	5.43***	0	0	0	0	12.58***	3.29*	10.43***	0	6.60***	23.02***	5.96***	1.42	2.47	0	12.48***	7.45***	0	0	7.07***		0	1.44	0	15.32***	0	3.96***	0	1.52	0	7.55***	0	0	0	1.41	0	0	0.62	1.11	14.81***	0	0	9.74***	2.81*	2.32	1.34	3.10*	4.41***	0	3.20*	2.95*	7.22***	1.27
BAI1. Numbness or tingling	0	0	6.43***	0	0	0	0	9.97***	5.19***	6.87***	5.69***	4.45***	5.83***	7.11***	0	3.69**	0	8.18***	11.33***	0	11.00***	0	10.80***	4.70***	0	0	0	0.42	0	0		0	16.69***	0	4.79***	7.73***	5.23***	18.52***	6.36***	0	5.50***	13.48***	14.23***	8.72***	12.69***	5.57***	4.48***	4.86***	4.93***	3.89**	3.93***	0	0	0	0	0	0	0	0	0	0	0
BAI2. Feeling hot	0	0	0	0	0	0	0	0	0	0	0	0	0	0	0	0	0	0	0	0.11	0	0	0	0	0	10.60***	0	0	0	1.44	0		0	0.77	0	0	0	0	0	3.26*	0	0	0	0	0	0	0	0	0	18.76***	19.20***	0	0	0	0	0	0	0	0	0	0	0
BAI3. Wobbliness in legs	0	0	4.10***	0	0	0	0	14.50***	9.96***	8.50***	10.51***	8.06***	9.34***	7.22***	0	4.76***	0	11.11***	12.35***	2.23	12.43***	0	16.26***	7.87***	0	0	0.10	0.58	0	0	16.69***	0		0.39	7.60***	8.61***	6.89***	24.84***	12.21***	0	9.61***	26.77***	29.46***	13.33***	17.10***	8.82***	8.13***	6.22***	11.49***	4.69***	5.46***	0	0	0	0	0	0	0	0	0	0	0
BAI4. Unable to relax	0	0	0	0	12.77***	15.33***	22.74***	0	0	6.86***	0	0	0	0	13.31***	3.89**	10.81***	0	4.72***	10.66***	6.56***	0	0	7.13***	0	5.99***	2.27	0	10.39***	15.32***	0	0.77	0.39		6.69***	3.24*	2.27	0	0	22.69***	0	0	0	8.52***	0	0	8.18***	1.48	10.29***	0	0	13.80***	9.34***	8.60***	7.24***	10.35***	10.38***	1.35	9.19***	8.92***	15.66***	5.79***
BAI5. Fear of worst happening	0	0	0.80	0	8.78***	2.21	12.41***	7.13***	3.82**	15.12***	0.24	0	0	1.58	5.73***	14.62***	1.51	7.76***	12.27***	3.95***	12.88***	1.60	5.45***	12.43***	0	0	12.76***	0.92	0.93	0	4.79***	0	7.60***	6.69***		7.34***	10.88***	9.06***	14.93***	10.49***	2.62*	6.29***	8.29***	25.18***	8.39***	14.40***	30.07***	7.60***	10.36***	5.17***	5.30***	6.95***	3.77**	2.54	0.22	2.40	0.08	0	5.13***	0.71	8.97***	0
BAI6. Dizzy or light-headed	0	0	0	0	4.55***	0.93	5.26***	0	0	5.16***	0	0	0	0	5.78***	1.95	0	0	5.47***	7.51***	5.34***	2.05	1.43	3.47**	0	8.81***	8.85***	4.62***	0	3.96***	7.73***	0	8.61***	3.24*	7.34***		14.51***	16.26***	0	2.59*	0	3.57**	6.57***	9.67***	4.61***	0	8.87***	11.29***	19.06***	7.48***	7.43***	0	0	0	0	0	0	0	0	0	0	0
BAI7. Heart pounding/racing	0	0	0	0	2.10	0	5.63***	0	0	4.96***	0	0	0	0	1.38	3.43**	0	0	3.79**	3.10*	4.22***	0.59	0	6.56***	0	14.01***	8.81***	3.61**	0	0	5.23***	0	6.89***	2.27	10.88***	14.51***		6.86***	1.17	5.51***	0	4.74***	6.60***	10.47***	6.98***	0	12.81***	9.76***	9.64***	11.83***	10.98***	0	0	0	0	0	0	0	0	0	0	0
BAI8. Unsteady	0	0	6.02***	0	0	0	0	10.23***	8.73***	9.99***	6.71***	3.83**	5.85***	5.74***	0.41	7.12***	0	8.93***	14.74***	4.67***	14.70***	0	14.46***	6.68***	0	0	1.21	0.63	0	1.52	18.52***	0	24.84***	0	9.06***	16.26***	6.86***		8.41***	0	5.68***	17.26***	20.27***	14.20***	14.84***	5.93***	9.38***	6.67***	14.77***	4.68***	4.60***	0	0	0	0	0	0	0	0	0	0	0
BAI9. Terrified or afraid	0	0	0.93	0	0	0	0	15.68***	6.11***	5.00***	20.27***	13.70***	10.57***	5.18***	0	3.87**	0	14.87***	7.48***	0	7.59***	0	14.58***	0.31	0	0	2.40	0	0	0	6.36***	0	12.21***	0	14.93***	0	1.17	8.41***		0	29.20***	14.17***	17.37***	13.62***	18.10***	28.16***	14.52***	0	0	0	0	0	0	0	0	0	0	0	0	0	0	0
BAI10. Nervous	0	0	0	0	12.32***	13.36***	26.99***	0	0	5.85***	0	0	0	0	11.00***	4.23***	9.34***	0	1.26	8.17***	2.01	0	0	6.02***	0	7.90***	8.78***	0	11.37***	7.55***	0	3.26*	0	22.69***	10.49***	2.59*	5.51***	0	0		0	0	0.83	10.30***	0	0	14.42***	2.04	8.14***	0.16	2.67*	12.26***	9.01***	7.96***	6.19***	9.25***	7.91***	1.38	8.19***	8.03***	15.06***	5.24***
BAI11. Feeling of choking	0	0	0	0	0	0	0	12.61***	3.51**	0.70	17.55***	12.61***	9.52***	4.55***	0	0	0	9.81***	4.81***	0	4.37***	0	12.12***	0	0	0	0	0	0	0	5.50***	0	9.61***	0	2.62*	0	0	5.68***	29.20***	0		11.07***	12.78***	4.40***	20.89***	18.88***	2.36	0	0	0	0	0	0	0	0	0	0	0	0	0	0	0
BAI12. Hands trembling	0	0	3.46**	0	0	0	0	16.07***	7.87***	6.79***	12.35***	8.94***	10.82***	6.02***	0	4.91***	0	12.72***	11.50***	0	11.37***	0	17.04***	4.32***	0	0	0	0	0	0	13.48***	0	26.77***	0	6.29***	3.57**	4.74***	17.26***	14.17***	0	11.07***		37.49***	11.65***	17.26***	10.19***	6.65***	3.27*	5.52***	2.41	3.52**	0	0	0	0	0	0	0	0	0	0	0
BAI13. Shaky/unsteady	0	0	4.65***	0	0	0	0	18.64***	8.75***	9.31***	14.35***	10.77***	12.80***	6.69***	0	6.00***	0	15.21***	13.07***	1.83	13.44***	0	19.52***	5.51***	0	0	1.05	0	0	0	14.23***	0	29.46***	0	8.29***	6.57***	6.60***	20.27***	17.37***	0.83	12.78***	37.49***		14.23***	20.75***	13.30***	9.51***	4.63***	8.81***	4.65***	4.99***	0	0	0	0	0	0	0	0	0	0	0
BAI14. Fear of losing control	0	0	1.50	0	7.69***	2.91*	11.71***	10.60***	6.91***	17.98***	3.87**	2.14	2.28	3.25*	6.96***	16.69***	0	9.06***	17.19***	5.72***	17.14***	0.10	10.53***	12.36***	0	0	13.46***	1.20	2.81*	1.41	8.72***	0	13.33***	8.52***	25.18***	9.67***	10.47***	14.20***	13.62***	10.30***	4.40***	11.65***	14.23***		10.79***	7.55***	26.19***	7.30***	13.40***	5.93***	6.81***	5.53***	2.44	0.26	0	1.48	1.92	0	2.77*	0	5.10***	0
BAI15. Difficulty breathing	0	0	3.11*	0	0	0	0	18.56***	8.38***	6.75***	12.62***	12.76***	12.03***	7.80***	0	3.54**	0	15.67***	10.45***	0.04	11.04***	0	19.60***	3.02*	0	0	0	0	0	0	12.69***	0	17.10***	0	8.39***	4.61***	6.98***	14.84***	18.10***	0	20.89***	17.26***	20.75***	10.79***		17.14***	7.51***	4.20***	5.00***	2.99*	1.59	0	0	0	0	0	0	0	0	0	0	0
BAI16. Fear of dying	0	0	0.67	0	0	0	0	11.73***	5.10***	1.06	15.04***	12.03***	9.77***	3.84**	0	0.09	0	16.76***	4.07***	0	3.71**	0	11.04***	0	0	0	0	0	0	0	5.57***	0	8.82***	0	14.40***	0	0	5.93***	28.16***	0	18.88***	10.19***	13.30***	7.55***	17.14***		9.72***	0	0	0	0	0	0	0	0	0	0	0	0	0	0	0
BAI17. Scared	0	0	0	0	10.06***	1.84	16.60***	7.51***	4.18***	16.91***	0.11	0	0	0.42	6.91***	14.54***	3.18*	7.71***	12.54***	5.64***	14.19***	2.02	5.06***	13.11***	0	0.20	18.69***	2.33	2.86*	0.62	4.48***	0	8.13***	8.18***	30.07***	8.87***	12.81***	9.38***	14.52***	14.42***	2.36	6.65***	9.51***	26.19***	7.51***	9.72***		9.58***	12.44***	6.41***	8.22***	6.96***	2.83*	1.35	0.92	2.00	0.16	0	4.97***	0.04	8.45***	0
BAI18. Indigestion	0	0	0	0	0.90	0	2.79*	0	0.03	5.78***	0	0	0	0	2.42	2.55	0	0	5.97***	4.94***	5.02***	0	0.49	5.78***	0	1.58	3.46**	17.34***	0	1.11	4.86***	0	6.22***	1.48	7.60***	11.29***	9.76***	6.67***	0	2.04	0	3.27*	4.63***	7.30***	4.20***	0	9.58***		14.54***	10.21***	6.96***	0	0	0	0	0	0	0	0	0	0	0
BAI19. Faint/light-headed	1.35	0	0	0	13.47***	7.66***	10.58***	4.70***	1.73	13.37***	0	0	0	0	13.29***	9.98***	3.75**	1.29	13.36***	18.22***	12.28***	4.68***	6.40***	8.82***	0	3.96***	8.01***	7.20***	3.22*	14.81***	4.93***	0	11.49***	10.29***	10.36***	19.06***	9.64***	14.77***	0	8.14***	0	5.52***	8.81***	13.40***	5.00***	0	12.44***	14.54***		8.78***	9.08***	5.72***	0.64	0	0	0.23	1.30	0	1.33	0	4.94***	0
BAI20. Face flushed	0	0	0	0	0	0	0	0	0	4.25***	0	0	0	0	0	0.32	0	0	1.21	1.08	1.30	0.20	0	4.19***	0	5.99***	3.81**	4.49***	0	0	3.89**	18.76***	4.69***	0	5.17***	7.48***	11.83***	4.68***	0	0.16	0	2.41	4.65***	5.93***	2.99*	0	6.41***	10.21***	8.78***		24.72***	0	0	0	0	0	0	0	0	0	0	0
BAI21. Hot/cold sweats	0	0	0	0	1.04	0	1.42	0	0	6.84***	0	0	0	0	1.87	2.50	0	0	3.99***	4.27***	4.27***	0.12	0.57	4.81***	0	11.09***	4.38***	4.23***	0	0	3.93***	19.20***	5.46***	0	5.30***	7.43***	10.98***	4.60***	0	2.67*	0	3.52**	4.99***	6.81***	1.59	0	8.22***	6.96***	9.08***	24.72***		0	0	0	0	0	0	0	0	0	0	0
W1. Overwhelming worries	0	0	0	0	13.11***	9.28***	15.64***	0	0	6.57***	0	0	0	0	9.98***	6.05***	13.64***	0	2.73*	6.61***	4.22***	0	0	0	0	1.23	0.23	0	7.95***	9.74***	0	0	0	13.80***	6.95***	0	0	0	0	12.26***	0	0	0	5.53***	0	0	6.96***	0	5.72***	0	0		22.89***	21.24***	14.59***	21.20***	17.72***	10.31***	20.65***	19.92***	25.55***	13.30***
W2. Situational worries	0	0	0	0	7.49***	3.91***	10.83***	0	0	3.92***	0	0	0	0	4.77***	4.60***	6.74***	0	0.57	0.62	1.13	0	0	0	0	0	0	0	5.62***	2.81*	0	0	0	9.34***	3.77**	0	0	0	0	9.01***	0	0	0	2.44	0	0	2.83*	0	0.64	0	0	22.89***		32.31***	25.25***	28.36***	17.84***	20.03***	27.84***	24.29***	21.60***	18.14***
W3. No control over worrying	0	0	0	0	5.56***	3.07*	9.79***	0	0	2.34	0	0	0	0	3.65**	2.32	5.23***	0	0	0.11	0	0	0	0	0	0	0	0	3.49**	2.32	0	0	0	8.60***	2.54	0	0	0	0	7.96***	0	0	0	0.26	0	0	1.35	0	0	0	0	21.24***	32.31***		23.17***	26.53***	16.21***	19.68***	28.54***	25.25***	21.96***	17.39***
W4. worrying under pressure	0	0	0	0	2.66*	1.52	7.45***	0	0	0.33	0	0	0	0	2.72*	0.71	1.15	0	0	0	0	0	0	0	0	0	0	0	3.01*	1.34	0	0	0	7.24***	0.22	0	0	0	0	6.19***	0	0	0	0	0	0	0.92	0	0	0	0	14.59***	25.25***	23.17***		21.69***	15.45***	17.04***	25.37***	21.03***	15.24***	16.18***
W5. Always worrying	0	0	0	0	8.42***	6.20***	11.93***	0	0	3.43**	0	0	0	0	5.48***	4.41***	7.67***	0	0	0.30	0	0	0	0	0	0	0	0	4.34***	3.10*	0	0	0	10.35***	2.40	0	0	0	0	9.25***	0	0	0	1.48	0	0	2.00	0	0.23	0	0	21.20***	28.36***	26.53***	21.69***		25.50***	24.79***	24.28***	29.90***	27.45***	19.55***
W6. To-do worries	0	0	0	0	6.50***	5.97***	9.47***	0	0	0.89	0	0	0	0	5.89***	3.19*	4.82***	0	0	1.41	0	0	0	0	0	0	0	0	4.70***	4.41***	0	0	0	10.38***	0.08	0	0	0	0	7.91***	0	0	0	1.92	0	0	0.16	0	1.30	0	0	17.72***	17.84***	16.21***	15.45***	25.50***		16.24***	17.16***	20.81***	21.40***	28.06***
W7. Lifetime worrier	0	0	0	0	0	0	2.79*	0	0	0	0	0	0	0	0	0	0	0	0	0	0	0	0	0	0	0	0	0	0	0	0	0	0	1.35	0	0	0	0	0	1.38	0	0	0	0	0	0	0	0	0	0	0	10.31***	20.03***	19.68***	17.04***	24.79***	16.24***		19.90***	22.31***	14.81***	15.09***
W8. Noticing worrying	0	0	0	0	6.95***	4.04***	10.75***	0	0	3.58**	0	0	0	0	4.12***	4.18***	5.53***	0	0.15	0.71	0.28	0	0	0	0	0	0	0	4.09***	3.20*	0	0	0	9.19***	5.13***	0	0	0	0	8.19***	0	0	0	2.77*	0	0	4.97***	0	1.33	0	0	20.65***	27.84***	28.54***	25.37***	24.28***	17.16***	19.90***		22.89***	23.80***	18.57***
W9. Can't stop worrying	0	0	0	0	6.24***	4.62***	9.27***	0	0	1.26	0	0	0	0	4.66***	1.64	6.08***	0	0	0.02	0	0	0	0	0	0	0	0	3.70**	2.95*	0	0	0	8.92***	0.71	0	0	0	0	8.03***	0	0	0	0	0	0	0.04	0	0	0	0	19.92***	24.29***	25.25***	21.03***	29.90***	20.81***	22.31***	22.89***		25.93***	18.07***
W10. Constant worrying	0	0	0	0	13.92***	10.83***	18.35***	0	0	6.71***	0	0	0	0	9.65***	7.91***	11.79***	0	2.37	5.56***	4.11***	0	0	1.27	0	2.37	0.93	0	8.31***	7.22***	0	0	0	15.66***	8.97***	0	0	0	0	15.06***	0	0	0	5.10***	0	0	8.45***	0	4.94***	0	0	25.55***	21.60***	21.96***	15.24***	27.45***	21.40***	14.81***	23.80***	25.93***		17.21***
W11. Worry about projects	0	0	0	0	2.17	1.21	6.19***	0	0	0	0	0	0	0	3.01*	2.07	1.13	0	0	0	0	0	0	0	0	0	0	0	1.12	1.27	0	0	0	5.79***	0	0	0	0	0	5.24***	0	0	0	0	0	0	0	0	0	0	0	13.30***	18.14***	17.39***	16.18***	19.55***	28.06***	15.09***	18.57***	18.07***	17.21***	

The effect size (t-value) represents the difference in the average distance between the pair of items compared to the distance between all other items.
A zero indicates that the mean distance between the pair of items is greater than the distance between all other items.
***: p<0.01; **: p<0.001; ***: p<0.0001**

Supplementary Table 2: Baseline characteristics and sum scores of the total sample, and the included and non-included subjects separately

		Total sample (n=2981)	Included in analyses (n=1649)	Not included in analyses (n=1332)	p-value
**Demographic characteristics**					
Age at baseline in years (mean; SD)		41.8 (13.1)	41.5 (12.7)	42.1 (13.4)	0.204
Female (%)		66.4	68.7	64.7	0.022
Level of education (%)					
	Basic	6.7	3.3	9.2	<0.001
	Intermediate	58.2	57.6	58.7	0.517
	High	35.1	39.1	32.0	<0.001
**Baseline scores**					
	IDS (mean, SD)	21.5 (14.1)	22.0 (13.0)	21.1 (14.9)	0.069
	BAI (mean, SD)	12.1 (10.7)	11.9 (9.4)	12.3 (11.6)	0.281
	PSWQ (mean, SD)	30.8 (11.9)	32.5 (11.1)	29.1 (12.5)	<0.001
