# Supplementary material for: Unveiling Transitions in Disease States: Study of Depressive and Anxiety Symptom Networks over Time
Source: Depress Anxiety. 2024 Jul 16;2024:4393070. doi: 10.1155/2024/4393070 (PMC11918905; doi:10.1155/2024/4393070)
Supplement: Supplementary 2 — Results of the generalized linear model of repeated measures of sum scores over time of (a) the PSWQ, (b) the BAI, and (c) the IDS for the diagnostic subgroups showing the overall F-value of between-subjects contrast, mean difference (MD) and standard error (SE). [file 4393070.f2.docx]

**Table Additional file 1**: Results of the generalized linear model of repeated measures of sum scores over time of [A]: the PSWQ, [B]: the BAI and [C]: the IDS for the diagnostic subgroups, showing the overall F-value of between-subjects contrast, mean difference (MD) and standard error (SE)

**[A] PSWQ**

| Overall F-value of between-subjects contrast: 248.735 (p<.001) | | | | |
| --- | --- | --- | --- | --- |
|  | **Controls  (n=360)** | **MDD-only  (n=265)** | **AD-only  (n=158)** | **AD-MDD comorbidity (n=866)** |
| **Controls  (n=360)** |  | MD: -6.010***  SE: .725 | MD: -7.706***  SE: .841 | MD: -14.763***  SE: .564 |
| **MDD-only (n=265)** | MD: 6.010***  SE: .725 |  | MD: -1.696  SE: .886 | MD: -8.753***  SE: .628  P <.001 |
| **AD-only  (n=158)** | MD: 7.706***  SE: .841 | MD: 1.696  SE: .886 |  | MD: -7.057***  SE:.760 |
| **AD-MDD comorbidity (n=866)** | MD: 14.763***  SE: .564 | MD: 8.753***  SE: .628 | MD: 7.057***  SE:.760 |  |

**[B] BAI**

| Overall F-value of between-subjects contrast: 73.308 (p<.001) | | | | |
| --- | --- | --- | --- | --- |
|  | **Controls  (n=360)** | **MDD-only (n=265)** | **AD-only  (n=158)** | **AD-MDD comorbidity (n=866)** |
| **Controls  (n=360)** |  | MD: -1.724  SE: .800 | MD: -3.289**  SE: .847 | MD: -7.589***  SE: .642 |
| **MDD-only (n=265)** | MD: 1.724  SE: .800 |  | MD: -1.565  SE: .810 | MD: -5.865***  SE: .592 |
| **AD-only  (n=158)** | MD: 3.289**  SE: .847 | MD: 1.565  SE: .810 |  | MD: -4.300***  SE: .655 |
| **AD-MDD comorbidity (n=866)** | MD: 7.589***  SE: .642 | MD: 5.865***  SE: .592 | MD: 4.300***  SE: .655 |  |

**[C] IDS**

| Overall F-value of between-subjects contrast: 198.143 (p<.001) | | | | |
| --- | --- | --- | --- | --- |
|  | **Controls  (n=360)** | **MDD-only (n=265)** | **AD-only  (n=158)** | **AD-MDD comorbidity (n=866)** |
| **Controls  (n=360)** |  | MD: -5.446***  SE: .790 | MD: -5.149***  SE: .899 | MD: -13.895***  SE: .627 |
| **MDD-only (n=265)** | MD: 5.446***  SE: .790 |  | MD: .298  SE: .912 | MD: -8.449***  SE: .646 |
| **AD-only  (n=158)** | MD: 5.149***  SE: .899 | MD: -.298  SE: .912 |  | MD: -8.747***  SE: .775 |
| **AD-MDD comorbidity (n=866)** | MD: 13.895***  SE: .627 | MD: 8.449***  SE: .646 | MD: -8.747***  SE: .775 |  |

* p<.05, ** p<.01, *** p<.001
