# Supplementary material for: Unveiling Transitions in Disease States: Study of Depressive and Anxiety Symptom Networks over Time
Source: Depress Anxiety. 2024 Jul 16;2024:4393070. doi: 10.1155/2024/4393070 (PMC11918905; doi:10.1155/2024/4393070)
Supplement: Supplementary 3 — Results of the generalized linear model of repeated measures of sum scores over time of the PSWQ, the BAI, and the IDS comparing subsequent disease state stability scores, showing the overall F-value of between-subjects contrast, mean difference (MD), and standard error (SE). [file 4393070.f3.docx]

**Additional file 2:** Results of the generalized linear model of repeated measures of sum scores over time of the PSWQ, the BAI and the IDS comparing subsequent disease state stability scores, showing the overall F-value of between-subjects contrast, mean difference (MD) and standard error (SE).

|  | | **PSWQ** | | | **BAI** | **IDS** |  |
| --- | --- | --- | --- | --- | --- | --- | --- |
| **Overall (F; p value)** | | F=259.386; p<.001 | | | F=121.802; p<.001 | F=307.712; p<.001 |  |
| **Post hoc sum scores comparisons (MD (SE))** | | | | | | |  |
|  | 0 vs 1 | | -5.62 (.574)*** | -1.54 (.643) | | -4.22 (.592)*** | |
|  | 1 vs 2 | | -3.89 (.627)*** | -2.59 (.574)*** | | -4.07 (.595)*** | |
|  | 2 vs 3 | | -4.82 (.681)*** | -1.78 (.582)*** | | -2.99 (.639)*** | |
|  | 3 vs 4 | | -.93 (.709) | -1.41 (.567)*** | | -3.48 (.663)*** | |
|  | 4 vs 5 | | -5.30 (.738)*** | -5.42 (.562)*** | | -7.89 (.680)*** | |

* p<.05, ** p<.01, *** p<.001
